# Taking control of one’s everyday life - a qualitative study of experiences described by participants in an occupational intervention

**DOI:** 10.1186/s12889-023-15515-z

**Published:** 2023-03-30

**Authors:** Louise Karlsson, Lena-Karin Erlandsson, Anna Cregård, Lena Nordgren, Marie Lydell

**Affiliations:** 1 Centre for Clinical Research Sörmland, Uppsala university Sweden, Region Sörmland, Gnesta, 64635 Sweden; 2 School of Health & Welfare, Halmstad University, Halmstad, Sweden; 3 Dept. of Work Life and Social Welfare, University of Borås, Borås, Sweden; 4 Dept. of Public Health and Caring Science, Uppsala University, Uppsala, Sweden

**Keywords:** Empowerment, Everyday life, Health, Home-related demands, Mental illness, Occupational balance, ReDO®, Stress, Work

## Abstract

**Background:**

Sick leave due to stress-related ill-health is increasing and is often caused by occupational imbalance. These types of issues tend to affect both the ability to work and cope with everyday life, as well as the overall experience of health, negatively. There is still little knowledge on how to prepare people and workplaces for the return-to-work process after participation in a work rehabilitation program due to stress and occupational ill-health. Therefore, this study aimed to describe what is needed to achieve a balanced everyday life that includes paid work as experienced by individuals who had participated in a ReDO® intervention due to occupational imbalance and ill-health.

**Methods:**

The concluding notes from 54 informants’ medical records were used for qualitative content analysis. The informants had participated in an occupational therapy group intervention to promote occupational health and regain full work capacity.

**Results:**

The analysis resulted in one major theme and four categories describing how the informants perceive that they must take control of their everyday life as a whole. By doing so, they need to work with structurization and prioritization, social interaction, boundary setting, and occupational meaningfulness.

**Conclusion:**

The study indicates a highly relational process, where it is impossible to divide life into private and work, and presupposes balance in everyday life in multiple dimensions. Its contribution includes the formulation of perceived needs in the transition between intervention and return to work and could, through further research, be used to generate a more effective and sustainable return- and rehabilitation models.

## Background

Work is an important part of health. One study shows that individuals with paid work rated their overall health 10% higher than the unemployed, and 24% more of those unemployed experience issues with anxiety and depression [1]. According to a Swedish report from 2018, almost a third of the employed work force in Sweden has suffered from work-related stress during some part of their life, something that seems to be increasing in other countries such as e.g., the UK [2, 3]. The costs associated with different sorts of stress related illness for individuals, employers and overall society are high. Apart from its impact on individual’s well-being, the total costs of mental ill-health are estimated to exceed EUR 600 billion – or more than 4% of GDP – across the 28 EU countries [4]. Mental illness, including stress-related ill-health, continues to be the largest cause of sick leave in Sweden, according to the Swedish Social Insurance Agency [5]. The WHO has estimated that it is becoming one of the greatest and fastest-growing public health challenges globally [6, 7]. Already in 2009, the European Commission estimated that 50–60% of lost working days in the European Union were linked to work-related stress [8].

The term occupation is most often equal to paid work or a profession; however, in this study the word occupation refers to an individual doing something in an environment in any daily area, such as paid work, schooling, household chores, rest or leisure [9]. Occupational balance is here thus the balance between all different occupations in everyday life and is construed as the extent to which an individual’s unique occupational patterns (in context) enable needs essential for well-being and quality of life [9, 10]. Occupational patterns are created when a person does things over time [10]. Occupational balance is associated with both perceived health and wellbeing [11] as well as ability to work [12, 13]. In addition to sleep, paid work is the area of occupation where adults spend most of their time and which affects their occupational patterns to the greatest extent [14]. Paid work is a crucial part of everyday life, and its impact on the perception of occupational balance can be a source of both health and ill-health [14, 15]. For the individual, the occupations related to paid work have a multidimensional value both personally and socioculturally [9]. Accordingly, if an individual is unable to work due to, for example, stress, occupational ill-health can be caused by the roles and habits that change when on sick leave through exclusion from the social context at work [12, 13].

Thus, if a person’s participation in daily occupations does not correspond to his or her unique physical, social, and mental needs, a state of occupational imbalance occurs. Occupational imbalance and the corresponding occupational balance always refer to subjective experiences [12] depending on the individual’s e.g., capacity, challenges and, resources in his or her everyday life. This state can precede the state of occupational ill-health. To achieve, or regain, health, the person needs to be able to do what they want and need in the environment that he or she is in [12]. Based on these core conceptions stated by A. Wilcock, the ValMO-theory [16, 17], and research on occupational patterns and health, an occupational therapy group intervention was developed. The intervention – named Redesigning Daily Occupations (ReDO®) – aims to support individuals with occupational ill-health through different tools and strategies in order to create and maintain occupational balance and health with time for paid work [18]. The ReDO® intervention was originally developed as an occupational therapy group intervention for women with stress-related disorders, and the intervention programme is based on studies of women’s daily occupations and their relationship to health. ReDO® addresses the whole occupational pattern 24 − 7, including paid work[19–21]. The ReDO® intervention in its original form consists of ten weeks of group sessions, seminars, and homework followed by six weeks of work placement [18, 22]. The ReDO-16 has in several studies shown a good outcome effect[21, 22], including from a longitudinal perspective [23]. The original ReDO® -16 programme has been adapted to a shorter, ten-week programme, the ReDO®-10. The results from evaluation of the ReDO® -10 show similar outcome effects as the original, at end of treatment and at six months follow-up. There was significant improvement in perceived health, occupational balance, occupational values and sense of mastery [24]. The inclusion criteria for participating in a ReDO® intervention were that the individual should be on, or at risk of developing, sick leave, i.e., being of working age and experiencing one or more of the following problems: (a) a high frequency of health care visits, (b) complex symptoms, (c) pain with varied diagnoses or symptoms, (d) mental illness that can be treated in a primary care setting or (e) other symptoms/disorders resulting in a perceived occupational imbalance. The ReDO® intervention is manually driven, and all ReDO® interventions are led by occupational therapists (OTs) certified as ReDO® leaders. This means that all ReDO® groups follow the same set of sessions in a chronologic order with the same content. After the last session the intervention ends with the treating OT writing a concluding note in the informant’s medical records. The concluding note summarises the participating individuals’ needs, achievements and strategies based on the individuals’ own words. This is often collected verbally through an individual meeting, or within the group. The participating individuals are asked open questions considering how they experience their everyday life, if there have been any changes, and what they have learned and want to continue to work with by themselves. Thus, the concluding notes are not the treating OT’s interpretations of the patient’s experience; it is a conclusion of the patient’s opinion of his/her situation at the end of the intervention.

Due to the cost of mental illness and stress related ill-health for society and employers, many policy makers view workplace mental health as a major issue and seek interventions that may be effective in preventing mental illness related to the workplace. There is limited consensus about the work-related effectiveness of various mental health interventions [25]. However, finding recovery during work hours seems to have a health promoting effect and can involve small things such as listening to music, laughing with a co-worker or going for a walk during a break [26, 27]. Nevertheless, having the opportunity to decide for yourself which occupation can assist in your recovery seems just as important [28]. Another review that examines workplace interventions to support mental health and well-being stated that it is important that the employees feel engaged and involved in the development of the intervention and implementation process [29].

Interventions often include a process for change, which can be explained through the elements of the personal empowerment process, i.e., experiencing powerlessness, gaining awareness, learning new roles, initiating participation and contribution are important [30]. An intervention can provide knowledge and tools needed for health behaviour change [31]. However, to change habits and routines in everyday life is not easy, [32, 33] and in the process of change, empowerment can play a key role in success [34]. Empowerment has several different meanings in different areas of knowledge, but in this study, it is defined as the individual process that enhances a person’s self-esteem. It focuses on their possibilities to make their own choices, and the conditions and resources required for them to become aware of and make rational decisions in different situations as empowerment through occupation and participation in society [35].

Although there has been an increase in mental health promotion and prevention programmes globally, only 7% of such initiatives are workplace-based [36]. According to OECD, mental health at the workplace is considered a future challenge for the labour market that needs to be prioritised [4]. A recent review concluded that more insight in facilitating factors and barriers for the implementation of worksite health that promotes intervention is needed in order to know which practical toolkit is most effective [37]. It has also been shown that home-related demands and resources influence the return-to-work-process among individuals sick-listed for common mental disorders. This also needs to be considered, even when work-related demands are experienced as the main reason for the sick leave period [38].

Thus, there is a lack of knowledge of the return-to-work process that takes everyday life, including both work and home-related demands, into consideration. This knowledge is needed to create a better understanding of the area and adapt existing interventions or create new ones that meet the demands to promote health.

The aim of this study was to describe the needs to achieve a balanced everyday life with paid work included, experienced by individuals who had participated in a ReDO® intervention due to occupational imbalance and ill-health.

## Methods

### Design and settings

This study used a qualitative research design with an inductive approach. The data are constituted of the concluding notes kept in the medical records for informants who had undergone the ReDO® -intervention. The data collection for the study was conducted from March 2021 through August 2021, in eight different regions in Sweden.

### Data collection

To gain access to the concluding notes, all OTs trained in the ReDO® intervention in Sweden were contacted. The OTs were listed at the Swedish Association of OTs, and approximately 300 individuals were contacted and asked to assist with the withdrawal of data from medical records. The OTs were identified through a digital platform dedicated to certified ReDO® -group leaders. Emails were sent out in July 2020, January 2021 and February 2021 through the digital platform, and a final group of 12 OTs from different parts of Sweden joined to help with the data collection. The included OTs all had held at least one ReDO® group from 2016 to 2021 and had written concluding notes from the intervention that were kept in their informants’ medical records. Both the OTs and their employer were invited to a digital meeting to acquire more information about the upcoming study, the aim, the data collection process and the expected time required. The digital meeting was held in March 2021 with a follow up meeting in April 2021.

**Fig. 1:**
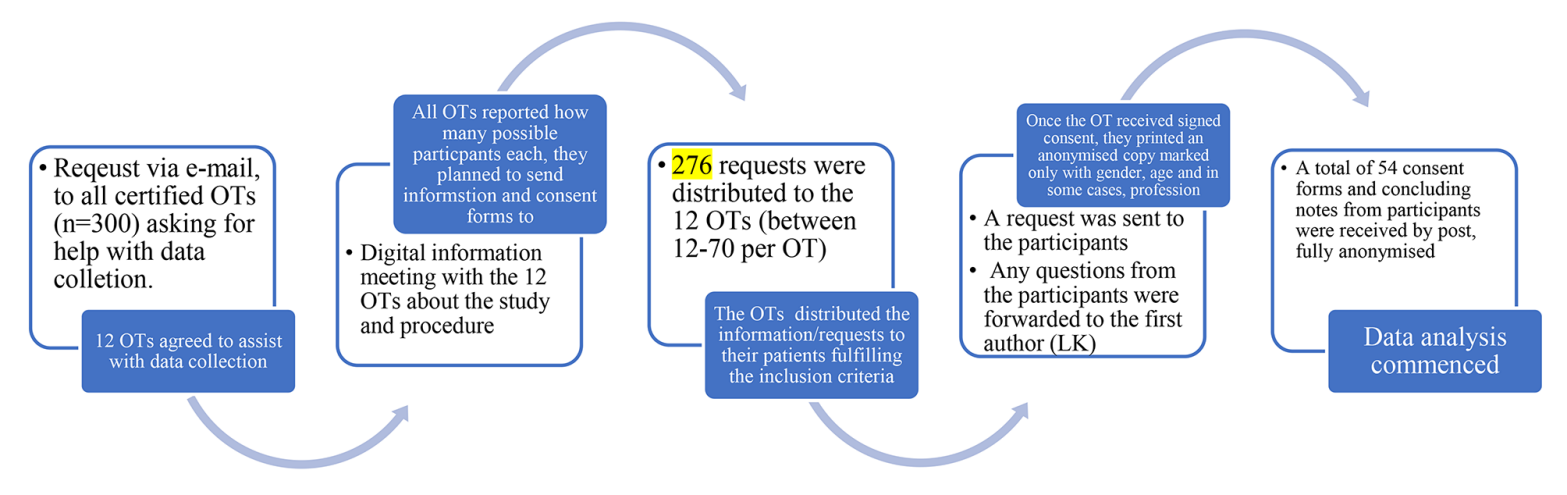
Data Collection process

Written information and consent forms were sent to the volunteering OTs in April 2021, who then forwarded them in May 2021 to informants who had participated in a ReDO® intervention between 2016 and 2021. A total of 276 potential informants had participated in the intervention during the period, and requests were sent out to all of them. Then signed consent forms were returned to the OT, who printed a deidentified copies of the concluding note from the informants’ medical record and sent them by post to the first author. To ensure that no connections could be made between the consent forms and concluding notes they were sent in different envelops, see Fig. 1.

Before the concluding notes were sent, they were coded by the OTs according to gender, age, year of the ReDO® intervention, and in some cases place of work if the informant chose to share that on the consent form (See Table 1). They were then sent to the first author between June – August 2021. A total of 54 concluding notes varying from a short note of three-four sentences up to three pages, with a median of one page, were included in this study.

### Informants

Informants were recruited through OTs at clinics where they had participated in a ReDO® intervention (n = 54 of 276). Informants who had participated in both ReDO-10 and 16 were included. Inclusion criteria for this study were that the informants should have finished their intervention between 2016 and 2021, be of working age, and have a final medical record from the intervention. The concluding notes from ten men aged 32–63 years, and 44 women aged 27–62, with a median of 46 years for both genders, were included in the study. For the distribution between gender and different areas of work, see Table 1.

**Table 1:** The informants’ current area of profession when giving their consent to participate in the study, in 2021

Current employment	n	w/m
Unemployed/	2	1/1
IT and office	14	10/4
Industry	4	4/0
Trade	3	3/0
Health care	17	16/1
School and childcare	11	8/3
Farming and gardening	3	2/1

### Data analysis

The concluding notes were analysed using inductive qualitive content analysis focusing on the manifest content [39]. The first author collected all 54 individual notes, approximately 60 pages. The reading started when all concluding notes were collected, and these were read thoroughly several times to create an overall image of the material. In the next step meaning units from selected texts that related to the objective of the study were identified, and extracted, by the first author. Some concluding notes contained different measures from before and after the intervention, which were not useful for this study, and therefore excluded. All the different meaning units were sorted based on their content and were then condensed and abstracted into codes. This was done by putting Post-it notes on a board. Similar codes were grouped into categories describing the content, and an overall theme was created that described all the different categories. The last author was included in every step and was given all the material as the analysis transpired, and together through reflection and discussion the different categories and the overall theme were set. The coding and tentative categories were discussed between mainly the first and last author, but all authors had access to the material during the process.

### Ethical considerations

Ethical approval for the study was granted by the Swedish Ethical Review Board (dnr 2021 − 01071). All informants received a letter with written information about the purpose of the study, the meaning of contribution, and that their anonymised concluding notes would be used for analysis on a group level. Information about confidentiality, free participation and that the informants could request to withdraw their documents from the study at any time was included in the letter. Written informed consent was then obtained from the informants.

## Results

The findings reflected the information extracted from the concluding notes of 54 individuals who had accomplished their experience of the need to create a more balanced everyday life with paid work included, after they had participated in a ReDO® intervention. In the presentation of the results, anonymous codes are used to protect the informants’ identities.

**Fig. 2:**
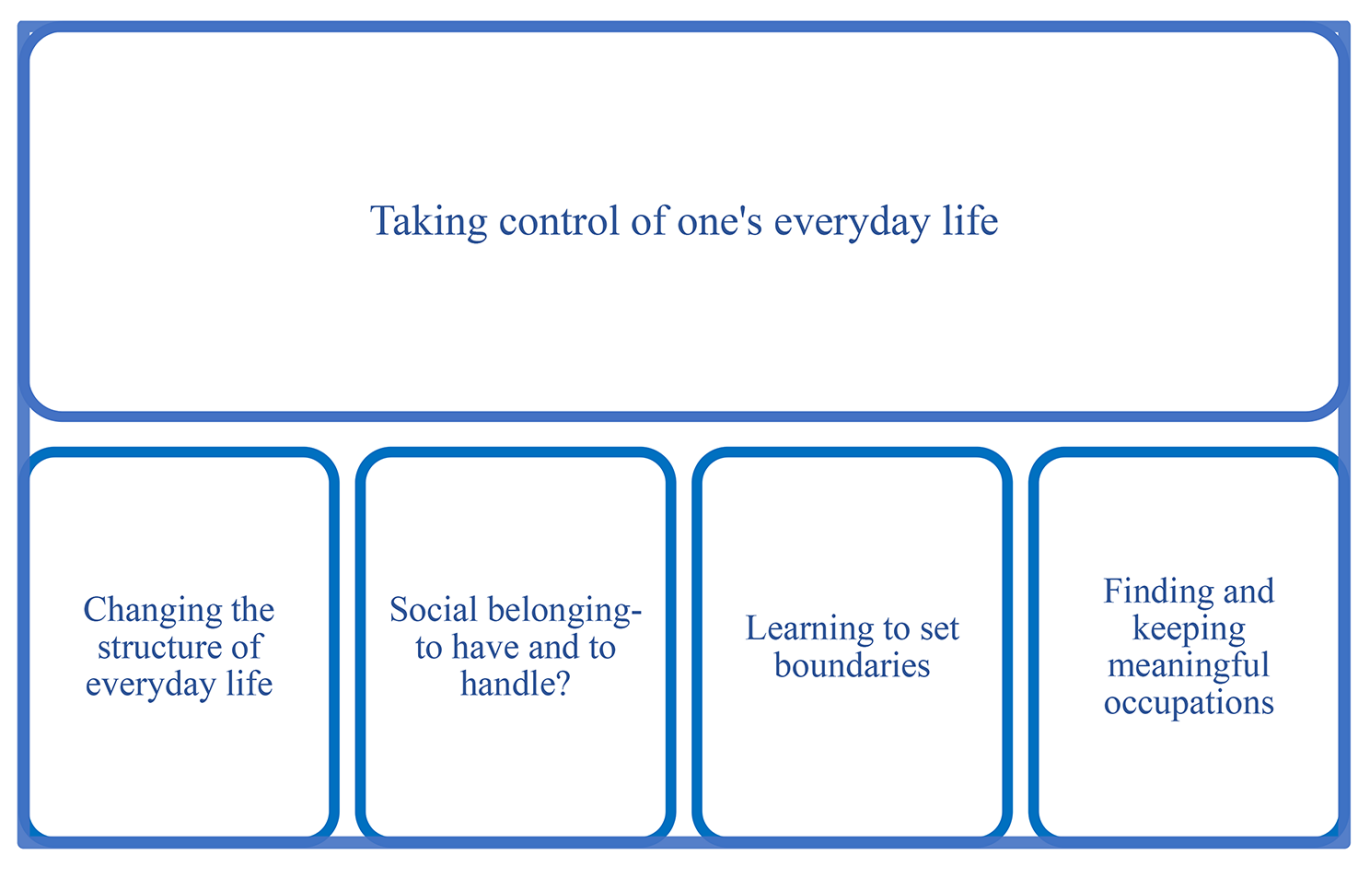
Results of the qualitative content analysis of (n 54) final notes regarding experienced needs when ending the intervention, showing the overall theme with the four main categories

The thematic findings were compiled within one overall theme: “Taking control of one’s everyday life”, further discussed below. Four main categories were created to capture the key aspects of the informants’ needs, see Fig. 2.

### The overall theme “ *Taking control of one’s everyday life”*

The overall theme - “Taking control of one’s everyday life” - captures the experiences of the informants’ needs in their strive for occupational balance in everyday life, as found in the concluding notes after the ReDO® intervention. It also described in what way the informants had started to change their occupational patterns in everyday life in different ways, such as how they value different things and the process of trying to build a new more balanced everyday life that promote their health. However, it also showed the struggle of breaking old habits and creating new ones with, or without, help from their social surroundings. The overall theme illuminates the needs in trying to find a balanced everyday life with time and energy for both work, home-related demands, and recovery.

### Changing the structure of everyday life

It was found that the part of the intervention that focused on the informants’ present, where they had to map their current everyday life to see what changes that needed to be done, was important. This category captured the informants’ new insights when it comes to their own needs to create balance in their everyday life through planning, prioritising, and performing occupations to find a structure that fits them and their health. The result also highlighted the importance of taking one thing at a time, to be able to both fully focus on their current occupation and prevent making mistakes, as well as avoid feeling unnecessary stress, which the informants tended to once they tried to do everything at once.

*“She realized that she can´t do everything, learned to focus on one thing at a time and to remind herself of this” (Woman number 25)*.

The results also displayed the struggle of trying to do one thing at a time, since it made some informants feel non effective, like they were not taking full advantage of their time, which in turn could make them feel stressed.

*“Stressed about not being able to take full advantage of the time, finds it difficult to just take one thing at a time” (Women number 32)*.

The result also described how the informants had tried, and in some cases succeeded, to create new routines in their everyday life to try and maintain a balance between the different occupations by making schedules and planning activities for the upcoming week, both for work and household chores, as well as rest and recovery. They described a new way of thinking about occupations, to try to make them feel less boring or heavy and to make their schedule feel less stressful. The informants tried to live in the present.

*“She plans more breaks between activities and thinks ‘I want to’ instead of ‘I have to’. What I do should be pleasurable. Starts living in the present” (Woman number 12)*.

However, there were also informants who described the struggle and difficulty in trying to change their daily routines, that it was an ongoing process that could create both stress and frustration. However, the process also included a willingness to change.

*“Nn is easily blocked and has difficulties getting routines in order. But she has a forward-thinking stance, despite the frustration that it is going slowly " (Woman number10)*.

In this category the informants also described the realisation that perhaps one of the reasons that they ended up with an occupational ill-health was because they were prioritising the wrong things in life, which in turn could make them feel more stressed and unhappy about their situation. This was an important new insight of their needs that could affect change in their everyday life. They also described that their energy went towards paid work, buying groceries and cleaning instead of towards occupations that made them feel good and gave them value. Moreover, they mentioned the importance of learning to include the family in the household chores and stop thinking that everything had to be “perfect”.

*“Nn now has a different use of time: Less time for cleaning, more time for the family. Not so thorough with everything, and lets her son in to help out more at home” (Women number 12)*.

The informants also described that they had come to the realisation that they needed to minimise their time on social media since it takes way too much time away from other things that are more important as well as causes greater stress.

*“Nn listens to the body more. Has also prioritized away things that is stressful such as social media " (Man number 40)*.

### Social belonging - to have and to handle

From the concluding notes the pros and cons about the informant’s social context and environment were described. This category also showed how the surroundings could be both an asset but also an obstacle in the process of trying to create a more balanced everyday life. In this category authorities are described, such as the role of employers, the Swedish Social Insurance Agency, and the Swedish employment agency, as well as the role of family, friends, and others. There were also things in the informants’ lives or surroundings over which they had no control, but which affected their everyday life, such as separation, the death of a relative or moving due to a partner’s new job.

The informants described the freedom, the support, and the acceptance they felt when in a group with others with similar problems. They all described the group setting as a very important factor in their change process, that they needed to meet others who did not judge them or make them feel like a failure. They also needed training to be in social situations again and feel empowered, so that they could manage in future.

*“The group has been important as nn describes that she needed to train to get out and hang out with other people, and that it was a freedom to hang out with others who understand” (Women number 38)*.

The importance of having support from others was explained as a factor to be able to make a change in everyday life. This involved having someone to ask for help or just to socialise with in order to avoid losing focus and stay motivated in the process of changing and promoting occupational health.

*“Has a friend who is a clear role model / inspiration who always makes a " to do " list for the upcoming year at New Year’s Eve (Women number 26)*.

There were, however, informants who described a lack of meaningful social context, and how the pandemic had made it harder for them to go out and engage in meaningful occupations where they usually met other individuals. The concluding notes described how important the social exchange was to the informants and their well-being.

*“Nn misses the choir, the water gymnastics and just socialising which is now difficult due to the pandemic” (Women number 43)*.

The informants also described a frustration and sadness associated with having a problem that did not show on the outside, as they struggled with a problem that both authorities and employers could question, but their own family, neighbours, or employers could not see since it did not show. The struggle involved trying to get better, gain more energy and have the ability to do things, while at the same time trying to explain and validate to the outside world that you do not function the way that you use to and have a greater need for recovery.

*“Nn says that she sometimes experiences a misunderstanding and experience of demands from her surroundings that he is unable to live up to, due to brain fatigue which isn´t visible to others” (Women number 1)*.

The informants also depict that finding a good way to communicate their situation, or their health, to others was difficult, and that the words often came out wrong and their surroundings misinterpreted what they were trying to say, which sometimes made them not wanting to talk at all.

*“Nn states that he needs to find better ways to communicate with his family and friends” (Man number 40)*.

### Learning to set boundaries

From the concluding notes it was found that the informants had to find new ways of setting boundaries both towards others and themselves. This included being able to say no when there is no time, or when they just do not want to do something, and ask for help, delegate, and trust in others’ ability in order to function and feel good. It also involved finding a sustainability in their changes, so they did not fall back into old habits.

The informants described the importance of setting boundaries and not being “online”, or available for others, at all times. They also highlighted setting clear boundaries ahead concerning what they can or cannot do to maintain their wellbeing.

*“He is not as accessible anymore. Now he turns off the phone or does not answer when it´s not necessary " (Man number 5)*.

The informants often described themselves as “yes-sayers” and people pleasers in the concluding notes as a key component that contributed to why they became ill. They also mentioned that finding the confidence to say no to things they do not want to do or do not have the time for has been very important.

*“Nn has become explicit in saying no, both at work and at home. At work she can close the door and ask people who disturb, to go out. At home, she can go into the office when she wants to be undisturbed " (Women number 27)*.

They also explained that sometimes they even had to say no to things that they really would like to do in order to sufficiently recover and cope with the next day.

*“Got better at saying no to things, sometimes even fun things to find time for recovery” (Women number 15)*.

The informants described the importance of learning how to delegate, since they often did everything by themselves, never allowing anyone to help, trying to make everything as perfect as they liked. They had realised that this is not a healthy way of living, and that they needed to include others and delegate in their everyday life to improve or maintain their health.

*“Trust more in others, at home it will be done by allowing others in the family to participate in household chores more and at work by trusting that colleagues do what they are supposed to do” (Women number 8)*.

The informants also said that by setting boundaries they could find time for themselves and explore what really matters and makes them happy, which was key to being there for others. Hence, it was described that the participating individuals needed to take care of themselves before helping others.

*“Has gained an increased awareness of his situation and has begun to value his own time, needs and what is important. … . has always done everything for everyone else’s and has now realized the importance and value of putting his own needs in focus” (Man number 42)*.

### Finding and keeping meaningful occupations

The concluding notes outlined the importance of having, or taking the time for, meaningful occupations in order to take care of one’s health and trying new things in life. In this category, leisure activities such as riding, dancing, photography, hunting, being in nature, going to the gym, meditation, etc., were mentioned. To find something that you like to do, and to take care of yourself and try to create healthy habits in everyday life, was noted as important.

The informants described a need to find occupations that were right for them, and that they could moderate based on their daily form, for example, an occupation that could be done in their own time and that they had the ability to adapt on their own, such as being in the woods, meditating or choosing a training that they themselves could control or set the moderation for, based on their energy.

*“Going to the gym and move around gives energy. When nn walks on a treadmill he can regulate pace and time, which fits him very well” (Man number 5)*.

However, the informants also described the problem of having a lack of motivation or energy to take care of themselves once they came home from their paid work, since they experienced that once they arrived home from work, they were completely exhausted and had no more force or energy to offer themselves or anything else.

*“Nn has difficulty motivating herself to physical exercise, feels that her energy is exhausted after work” (Women number 37)*.

## Discussion

The aim of this study was to describe the needs to achieve an experience of a balance in their everyday life with paid work included, for individuals who had participated in a work rehabilitation programme (ReDO®) due to occupational ill-health. In general, this study showed that it is important to take back, or for the first time feel in control over, one’s everyday life, and not just over the work situation, i.e., to strive for experienced occupational balance and promote health in everyday life. In this sense the result is not surprising, even if earlier research has focused mostly on the workplace and the return-to-work process and not everyday life (the combination of workplace time and maintenance and leisure time)[40–42]. However, Rofcanin et al. (2021) problematise how employers can work with everyday life for their employees [43].

A somewhat new perspective emerged, however, in concluding the categories that comprised the main theme. The result described conditions that the informants were concerned about before going back to work. They were aware of the need to create new structures in everyday life focusing on recovery, as they did not want to go back to their old habits. They understood that to strive for experienced occupational balance, they needed to start creating a new structure in their everyday life by focusing more on time for meaningful occupations and recovery and less on, e.g., keeping the household in perfect condition. This illustrates the importance for the employer to recognize broader types of rehabilitation programs that address the whole life situation, to promote health in the workplace consider the employees’ individual needs in their current everyday life instead of unilaterally concentrating on the working conditions [44].

Another important finding regarded social media and how stressful it can be. The final notes summarised that by going “offline” the informants could feel more relaxed and focus on what they needed. This is a new area and dimension to health and everyday life that needs to be considered, without the presumption that the relationship must be negative [45] since some can find e.g. the occupation of scrolling on their phone relaxing.

The results also showed the need to experience belonging and to have social relationships of some kind. The informants expressed the importance of feeling accepted and validated in their current situation, both in their health and rehabilitation process. This relates to what Wilcock and Hocking [12] points out as the foundation for phycological wellbeing and health. Furthermore, the impact of the social environment is highlighted as an important factor for health in social empowerment [9, 46]. The concluding notes showed the importance of being part of a social and meaningful context with others, to create a balanced everyday life and promote health. Occupations that reinforce experiences of belonging are in the ValMO model [17] described as occupations imprinted by socio-symbolic occupational value. A lack of this type of occupational value was found to be one of the main risk factors in developing occupational ill-health, after experiencing occupational imbalance in everyday life [47]. It is also stated in another study that among young individuals social support from friends, family, and colleagues/one’s employer, appears to be critical for a successful rehabilitation after the return to work after burnout, as well as gaining a sense of control over the recovery process [48]. Therefore, it seems important to find opportunities for social occupations and support in both family life and at workplaces as a step towards reducing the statistics for work-related stress and ill-health [2, 5]. It is known that if the employer and manager use a health-oriented leadership it buffers the employees’ health, and to be a supportive manager is a part of that process [49, 50].

The results demonstrated that the fear of being questioned by employers, administrators at insurance offices or friends could lead to additional stress or isolation. These findings are in line with previous research showing that, for example, the insurers’ actions can affect the outcome of occupational rehabilitation and promote health by ensuring a smooth process flow and making the individual feel acknowledged [51].

Moreover, the importance of the informants learning to say no emerged and built up their confidence, encouraging them to consider their own energy versus what they thought other individuals might expect from them. This finding is supported by research that examines the importance of reaching empowerment and learning new roles and a feeling of contributing [30]. The ReDO® intervention may have contributed to changed health behaviour in the informants [31] and empowered them to believe in themselves, as empowerment is very important in order to succeed [34].

Finally, the results showed the significance of being able to participate in meaningful occupations and have the energy to do so. The informants struggled with not having sufficient energy left for other occupations once they came home from work. This was a situation that, for some of the participants, was recognized as one reason for why they had initially developed occupational ill-health. In Sweden the norm is to work 40 h weeks until retirement that from 2026 will be at the age of 67 [52, 53]. However, studies have shown that the benefits of having reduced worktime with a preserved salary and more time for recovery, family and participation in meaningful occupations could reduce the risk of becoming sick due to stress and promote occupational balance [40, 54, 55]. These studies confirm the predominant assumption that a certain amount of time should be allocated to different categories of daily occupations. However, in reasoning on balance between work and non-work time [56] suggested that instead, the nature of the occupations in everyday life should be addressed. Their recommendation supports recognising the occupational value in all unique occupations in daily life [17] and not one-sided discriminate between work and non-work or work and meaningful occupations. However, it is shown that during the toddler years in dual gender families women tend to in sum have more paid and unpaid workhours than men. [57]. Women also tend to take a bigger responsibility for household work and caring for the children [58]. These gender inequalities could affect the opportunities for women to allocate time for and maintain meaningful occupations and may to some extent explain why middle-aged women with children are overrepresented in the sick leave rates [5, 59].

The results here illustrated that the informants had gained new knowledge and recognised the importance of every day making changes, and not only regarding the work situation. Another study also found that home-related demands, as well as resources, impacted the return-to-work process, although work-related demands were experienced as the main reason for the sick-leave period [38]. The categories of the main theme illuminated the importance of having control and being able to re-do the everyday occupational patterns by learning to prioritize, having the opportunities and energy to do meaningful occupations, and experiencing belonging in a social context where you are accepted and valued despite the current situation. This new everyday life perspective should be recognised by health care, insurance businesses and employers. This strengthens earlier research showing that health promotion interventions at work must also include an individual and personal aspect [60]. Based on this study, it seems important to recognise individuals that are trying to regain workability and make changes in their daily occupations by offering opportunities, time, and social support. Illustrating rehabilitation needs from this perspective, it would be interesting to further study whether these aspects are recognised by employers in promoting health among the employed as well in individual rehabilitation cases.

### Methodological considerations

Although almost 300 certified OTs were asked to help with the data collection, only around 40 had implemented and worked with the intervention on a regular basis, which explains the low number of OTs helping with the data collection. Only one request of participation was sent out to all 276 potential informants. Avoiding sending out reminders was a strategy mainly to make the study design feasible for the OTs that had volunteered to send out information, collect consents and compile the final notes before sending them to the first author. This could be a reason for the low response rate. Another reason may be that it has been some time since the informants participated in the intervention.

The data source in this study was documentation in medical records, reflecting the informants’ experiences of needs and strategies when ending the ReDO-programme. More and richer information in every single informant’s case could certainly be collected through, e.g., interviews. However, the final notes used in this study are part of the intervention programme, and all documenting OTs had followed the same steps in the manual to conclude the participants’ perception of their end-status. Thus, we consider the data used in this study relevant and reliable. Furthermore, the use of documentation in medical records did not burden the informants, which was considered important due to their continuing rehabilitation process.

The final notes are intended to capture the participants’ learnings in their everyday life after an occupational therapy intervention [18]. It would certainly have been beneficial for the study if the OTs had gathered more information regarding the participants’ working situation. However, the final notes from the ReDO-intervention, are based on the participants’ subjective experiences when ending the programme and the limited focus on their working situation reflects their view, at that occasion. Furthermore, not all informants were back to work when the final notes were written, which could therefore explain the lack of work-related reflections in the results. However, when the consent finally was collected, everyone except four informants reported being back to work either part- or full time.

All this notwithstanding, there is a risk that meaningful information might be lost in the process. Another challenge is that the occupational therapists wrote the final notes in very different ways: some wrote several pages, and some just wrote a few lines. With that said, in a few cases some of the short notes could contain more information than those consisting of several pages.

A qualitative content analysis was used to gain a further understanding and description of the informants’ experiences. The study aimed to stay close to the actual text of the final notes, applying an inductive, data driven thematic analysis [39]. Nevertheless, the researchers’ previous theoretical and clinical knowledge cannot be completely disregarded, but the theme of five authors coming from different scientific and empirical backgrounds ensures a broad perspective on both analysis and discussion.

The confirmability of the analysis was strengthened by using quotations in linking the data to the main concepts [61]. Though, the quotations illustrated the informants experiences, the analysis was close to the text. This strengthens the dependability. The first and last author were responsible for the open coding process, which strengthened the validation of this phase. To increase the trustworthiness of the study and to address the researchers’ preunderstanding, the interpretations and formulations of themes were discussed among all authors during the analysis process. The authors also discussed the findings in seminars with researchers in other research fields.

Furthermore, the included informants do reflect the average population of people on sick leave due to stress related ill-health in Swedish society, and the results may therefore be transferable for individuals of working age in Sweden [5].

## Conclusion

The overall result showed that even though the aim of an intervention might be to regain workability, it is still central or possibly even more important to first create and maintain an experience of balance in every day that promotes health and thereby one’s ability to work, too. The results of this study indicate that returning to work means a process that is highly relational, impossible to divide into private and work, and presupposes balance in everyday life in multiple dimensions. Its contribution includes the formulation of perceived needs in the transition between intervention and a return to work and regained health. Moreover, the result could, through further research, be used to generate more effective and sustainable work-return and -rehabilitation models. Further research is however needed concerning the sustainability and development of the changes over time as well as the employer’s point of view within the subject.

## Data Availability

The datasets generated and analysed during the current study are not publicly available due sensitive data such as medical records and cannot be freely shared. Even though the data is anonymised, the medical records contain information concerning specific situations or contexts that could pose a risk of recognition. However, data are available from the corresponding author and with the consent of the research participants on reasonable request.

## Abbreviations

WHO: World health organisation
OT: Occupational therapist
ReDO: Redesigning Daily Occupations
MoHO: Model of human occupation
